# Exploring well‐being: medical students and staff

**DOI:** 10.1111/tct.13080

**Published:** 2019-07-30

**Authors:** Victoria Simpson, Laura Halpin, Kirk Chalmers, Viktoria Joynes

**Affiliations:** ^1^ School of Medicine University of Liverpool Liverpool UK

## Abstract

**Background:**

Mental illness in young people is a major public health challenge, with a higher prevalence amongst medical students. This study explores the perspectives of both students and staff in relation to the provision of well‐being support within one medical school in the United Kingdom.

**Methods:**

A total of 17 second‐year medical students and five members of academic and well‐being staff at Liverpool Medical School participated in one‐to‐one semi‐structured interviews. Staff and students were asked for their views on existing support services, exploring challenges and ideas for development. Interviews were recorded, transcribed and analysed thematically to identify common themes amongst both students and staff.

**Results:**

Students wanted to have more obvious support during their transition from sixth form to undergraduate studies. Perceived stigma surrounding mental health continues to prevent students seeking help over concerns this might have upon academic progress. Staff reported concerns that student expectations did not always match with what could realistically be provided by the medical school well‐being service.

**Discussion:**

The provision of opportunities for students to ‘check‐in’ with staff, and the introduction of well‐being topics within the curriculum, were perceived by students and staff as being of potential benefit to the mental health of students. Such interventions may also help to build rapport and encourage students to engage with medical school support services. All well‐being support services are increasingly in demand amongst medical students, showing a need for them to expand and well‐being to be further incorporated into the course.

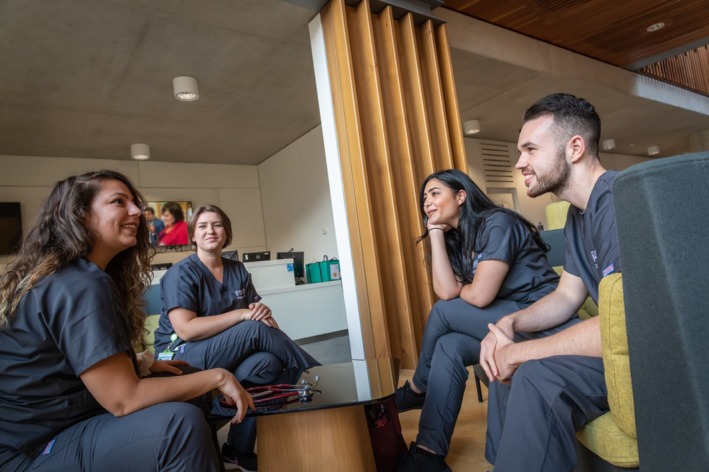

## Introduction

Adolescent mental illness is a rising public health challenge, and its prevalence is higher amongst medical students than age‐matched controls, with one study conducted in 2015 estimating that around 15% of United Kingdom (UK) medical students have experienced suicidal ideation.^1^ The demands of studying medicine, coupled with the adjustment necessary to uphold professional behaviours,^2^ are already known to impact student well‐being, with higher levels of stress reported in the earlier years of study.^3^ Previous research has identified that up to 80% of medical students feel under‐supported by their medical school,^1^ that admitting illness carries stigma^4^ and that medical students fear that seeking help could be detrimental to their training.^5^

Adolescent mental illness is a rising public health challenge, and its prevalence is higher amongst medical students than age‐matched controls …

This study aimed to investigate and compare the beliefs and experiences of staff and students at the University of Liverpool regarding medical student mental health. By exploring the views of students and staff, the study aimed to generate useful strategies for medical schools to provide support to students, within the confines of their primary remit as education providers.

By exploring the views of students and staff, the study aimed to generate useful strategies for medical schools to provide support to students …

## Methods

This study was conducted by three third‐year medical students (referred to here as the student researchers) as part of a student research project at the University of Liverpool School of Medicine (UK) that has a large (*n* = 300) yearly cohort, where a dedicated well‐being support service is available, with a focus on well‐being integrated into early curriculum content.

We conducted two phases of semi‐structured interviews. An experienced qualitative researcher (VJ) supervised both phases. Phase 1 (led by VS and KC) focused on exploring student experiences during their first year of study (perceived stressors, awareness and opinions of support services). Phase 2 (led by LH) focused on staff perceptions about their roles within medical student well‐being, the support services and their ideas for developing the service. The student researchers (VS, LH and KC) designed the interview guide and schedule with the precise wording and order agreed for both phases after discussions with the supervisor (VJ).

Second‐year medical students were recruited via social media pages available to all students; however we acknowledge that some students do not access social media frequently and therefore might have been excluded. Participants were in the academic year below the student researchers so that the interviews were not hindered by interviewees feeling that they were divulging personal information to close peers. Additionally, we advised participants that they could terminate the interviews at any time and appropriate support services were available to them if they became distressed when participating. Potential staff respondents were identified by LH and VJ, based upon the roles they held, and were invited to take part via e‐mail.

All interviews were audiorecorded, de‐identified and transcribed. For both phases we adopted a reflexive thematic analysis approach,^6^ with the student researchers (VS, LH and KC) re‐reading transcripts to code the data, and agreeing a final list of themes with the project supervisor (VJ). Only after this point did comparison of the results from each of the study phases occur.

Ethical approval for both phases was granted by the Liverpool University Faculty of Health and Life Sciences Research Ethics Committee.

## Results

A total of 17 second‐year medical students and five staff with roles in supporting medical students took part in the study. Five themes emerged from the study phases.

### Students adjusting to Year 1

In line with existing literature,^7^ students explained the transition to university provoked anxiety, especially in relation to what they perceived as a lack of academic guidance in comparison to sixth form level teaching. Students also reported feelings of ‘lacking validation’ from teaching staff that they were worthy of studying medicine (Table 1).

**Table 1:** Students adjusting to Year 1

Sub‐themes	Quotations
The jump from sixth form to university as a stressor	‘It's a big step from sixth form because you are expected to do your own work, it's all independent study and you don't get spoon fed’ (Student, Respondent 14)
Feeling unworthy of studying medicine	‘The first half of the year was a lot worse than the second half … It was an insidious build‐up of stress. Am I good enough to be here? … I felt like a fraud’ (Student, Respondent 15)
Student use of well‐being services	‘I went to student well‐being because … I was comparing myself to others … they went over my end of block test scores and said … you don't need to worry … I think that actually did help’ (Student, Respondent 3)
	‘I tried using the well‐being service to get the permission to leave for a funeral, which was granted without any problems’ (Student, Respondent 17)
	‘They made me a referral to move accommodation’ (Student, Respondent 4)

### Recognisable well‐being support

In addition to the curriculum content, the well‐being support that students identified included pastoral care from academic advisers and a specialist psychological support service for health science students that the medical school can signpost students to.

Students highlighted the benefits of designated time with staff members. Staff also acknowledged the importance of these interactions as potential opportunities to identify students in need of pastoral support. Staff recognised that students are allocated a new supervisor every 2 years, which could disrupt continuity of support. Staff also reflected that these meetings focused primarily upon academic progress, meaning that a student achieving well academically could slip under the radar from a well‐being perspective.

### Student misconceptions in relation to well‐being services

Many students interviewed had not attended well‐being services, but expressed apprehensions that reporting mental health concerns to the medical school might have negative consequences. Staff recognised that students did sometimes make inaccurate assumptions about seeking help, and felt that building closer relationships with students was one way to address this.

Staff also acknowledged that some students appeared to have unrealistic expectations of the university well‐being services – for example, in relation to staff expertise and availability. These unrealistic expectations were recognised by staff as putting a strain on the service, and meant some students were dissatisfied about not having had their perceived needs met (Table 2).

**Table 2:** Student misconceptions about seeking support

Sub‐themes	Quotations
Presumptions that reporting mental health concerns might have negative consequences	‘The medical school can say that we shouldn't feel afraid to ask for help but I'm afraid of being chewed out by admin and I don't want to deal with that when I'm already so worried about doing well’ (Student, Respondent 7)
	‘There is still a reluctance amongst medical students to come and talk to people because they think that it will affect their training’ (Staff, Respondent 2)
Dislike of discussion of academic progress during well‐being meetings	‘They were like “do you think this will impact on your studies, do you think this will impact on your future?” which shouldn't be the most important thing … I just want to chat with someone’ (Student, Respondent 2)
Perceptions of what well‐being services lacked	‘I think they didn't have as much experience of dealing with grief as they probably should’ (Student, Respondent 17)
	‘There should be a little bit more availability’ (Student, Respondent 6)

### Student preferences for seeking help

Staff perceived the demand for well‐being services as ‘increasing year on year’. Students expressed their own personal preferences for seeking help when they felt they needed it, highlighting there is unlikely to be an approach to providing support that suits all students. Students utilised a mix of formal support from the school. Staff reflected that they would value more time with students to build better relationships. Seeking support from peers was also favoured by many students, but this sometimes could result in further anxiety as some of these students appeared to compare themselves with their cohort (Table 3).

**Table 3:** Student preferences for seeking help

Sub‐themes	Quotations
Seeking help from other students	‘I don't think I would have survived first year if it wasn't for speaking to older students who helped me calm down’ (Student, Respondent 7)
	‘I had a second‐year mentor who was really helpful with things like how to study and where to get information from’ (Student, Respondent 16)
Comparing themselves to one another	‘If you hang around with medics, it's like “I know this, this and this” and it's scary’ (Student, Respondent 13)
	‘There's a few times where you're in lectures and you worry because they're so much cleverer than you’ (Student, Respondent 14)
Developing closer student–staff relationships	‘I think it would be great to develop relationships with students, to help them appreciate that we are kind of a family and we are here to look after them’ (Staff, Respondent 1)

### Further integration of well‐being into the curriculum

Staff discussed aspirations to integrate more well‐being into the medical curriculum to aid the welfare of students. Staff spoke about offering well‐being sessions including mindfulness and yoga to promote student relaxation and networking. However, the challenges of incorporating these sessions into a busy curriculum were noted by both students and staff (Table 4).

**Table 4:** Integration of well‐being into the curriculum

Sub‐themes	Quotations
The benefits of integrating more well‐being practice into the curriculum	‘We need to devote more time and energy to giving students coping skills, resilience training and to create a culture of being able to seek help from academics and peers’ (Staff, Respondent 4)
	‘We are considering what we can put in place to help develop people's positive mental health … to help them relax outside of the course’ (Staff, Respondent 1)
	‘… Meet people they haven't met before and find shared interests, enabling friendships and support networks to develop’ (Staff, Respondent 5)

## Discussion

A sense of diminished academic validation on entry to medical school was a widely reported stressor by students, and the foundation of some of the misperceptions of well‐being support.

A sense of diminished academic validation on entry to medical school was a widely reported stressor by students …

Despite a strong recognition of the importance of well‐being in both the formal curriculum and through support services, student respondents still mentioned a culture of ‘apprehension’ regarding seeking help, fearing that any involvement with well‐being or mental health teams would negatively impact their progression, in line with previous work in this area.^5^ Some students with adverse circumstances might decide to temporarily suspend their studies and be supported by the medical school. It is possible that some students perceive the idea of a temporary suspension as a ‘negative’ impact of seeking help, insofar as completion of their degree quickly is seen as paramount, and suspension is then seen as a ‘threat’ to this attainment. In practice, the school views supporting suspension on health grounds as a mechanism to ensure students have time to get well before being asked to resume their high‐pressured studies, and that studying medicine does not make them more unwell. Better management of the expectations of students (particularly those entering the medical programme direct from structured school environments)^7^ when they begin their degrees, regarding the remits of medical school support, could increase the utility of well‐being services. Students might be more forthcoming in seeking support if this ‘apprehension culture’ is challenged by the medical school through constant reinforcement of the value of well‐being, learning and student success.

Some non‐medical courses have smaller cohorts and fewer contact hours with staff, thus medical students spend more time completing work and being surrounded by peers. Interviewees identified both situations as sources of stress for medical students. It is increasingly common for all courses to have their own support services or at least access to centrally provided services, but given that medical students report amplified amounts of stress compared to other students,^2^ it is all the more paramount that tailored support services are available to them.

… medical students report amplified amounts of stress compared to other students, it is all the more paramount that tailored support services are available to them

Increased demand for all services, including referral to specialist psychological support, was noted. Staff stressed the importance of students being able to access this expert treatment provision (particularly when students are unwell), particularly at a time of shortfall in UK mental health support services. There is a real concern that students expect that well‐being services should be able to provide access to other services, such as secondary hospital care, but this is not always possible.

Managing these demands is particularly challenging with large student cohorts. Integrating well‐being more routinely into the curriculum (as also recommended by the General Medical Council^8^) represents a compromise, as it provides more opportunities for students to find a route to comfortably discuss personal circumstances as part of academic progress. Staff in this study were keen to integrate more well‐being options routinely into the programme (successfully implemented elsewhere^9^), which they hoped would enable students to build new friendships and support networks.

### Limitations

This study took place at a single site within the UK and therefore can make no claims to represent a wider population. The bespoke support services described are also context‐specific, the experiences of staff and students reflected here may be useful to those interested in supporting medical student well‐being.

## Conclusions

This study highlights that the transition to medical school remains an important cause of stress amongst students.^10^ The medical school in this study continues to develop materials for students at transition points including new study skills support, which will be evaluated.

This study highlights that the transition to medical school remains an important cause of stress amongst students

Optimising access to psychological support and the introduction of longitudinal support from staff were also recurring themes. A faculty‐wide review of access to the psychological support services is ongoing, and revisions to the academic adviser system to increase longitudinal contact with students will be introduced in the new academic year.

Nevertheless, students’ preconceptions about disclosing health or well‐being issues, and their unwillingness to seek help from formal medical school services, need to be addressed for interventions to be effective. For this small‐scale study, exploring and comparing the views of both staff and students has been incredibly informative, as it allowed to identify a mismatch between services provided and student expectations; such a methodology is recommended for future studies of this nature.

… students’ preconceptions about disclosing health or well‐being issues, and their unwillingness to seek help from formal medical school services, need to be addressed …

… it allowed to identify a mismatch between services provided and student expectations …
